# Supplementary Contribution of Eastern Cooperative Oncology Group-Performance Status to Quick Sequential Organ Failure Assessment in the Detection of Bacteremia Among Older Patients With Suspected Infections

**DOI:** 10.7759/cureus.55086

**Published:** 2024-02-27

**Authors:** Masataka Kudo, Sho Sasaki, Yu Yagi, Hiroshi Imura

**Affiliations:** 1 Department of General Internal Medicine, Iizuka Hospital, Iizuka, JPN; 2 Department of Nephrology, Iizuka Hospital, Iizuka, JPN

**Keywords:** eastern cooperative oncology group performance status, 30-day mortality, older patients, bacteremia, quick sequential organ failure assessment

## Abstract

Background

The Quick Sequential Organ Failure Assessment (qSOFA) is a simple method for identifying patients with bacteremia; however, it is not accurate for predicting it. Performance status assessment involves the evaluation of daily activities and could be beneficial in predicting bacteremia. We aimed to evaluate whether adding Eastern Cooperative Oncology Group-Performance Status (ECOG-PS) to qSOFA could improve the prediction of bacteremia diagnosis in older patients admitted with suspected infections.

Methods

Data were gathered from individuals aged ≥65 years who were hospitalized with suspected bacteremia from 2018 to 2019. Two prediction models were contrasted employing logistic regression. The initial model exclusively incorporated the qSOFA score, while the second model integrated the Eastern Cooperative Oncology Group-Performance Status (ECOG-PS) alongside the qSOFA score.

Results

Among 1,114 enrolled patients, 221 (19.8%) had true bacteremia. The area under the curve of the qSOFA+ECOG-PS model did not show a statistically significant improvement in predictive capacity compared with that of the qSOFA model (0.544 vs. 0.554, p=0.162).

Conclusions

Adding the ECOG-PS score did not improve the performance of qSOFA for predicting bacteremia in older patients with suspected infection.

## Introduction

Mortality caused by sepsis is high, reaching up to 11% in older patients [1,2]. Early identification and treatment of bacteremia can lower the mortality rate [3-5]. The 2016 Surviving Sepsis Campaign guidelines introduced the Quick Sequential (Sepsis-Related) Organ Failure Assessment (qSOFA) tool as an integral component of the revised definitions for sepsis and septic shock. Subsequent studies employing the qSOFA for bacteremia prediction have emerged, revealing that the sensitivity and specificity of qSOFA scores for bacteremia range between 23.0-47.0% and 61.8-91.0%, respectively, with an area under the receiver operating characteristic spanning 0.58-0.59 [6,7]. Consequently, its efficacy in predicting bacteremia is limited [8]. Nonetheless, the qSOFA score, a streamlined tool employing three vital signs, in conjunction with additional findings, may enhance the precision of bacteremia prediction. The combination of a qSOFA score of 2 or higher plus a lactate level of ≥2 mmol/L exhibited a receiver operating characteristic (ROC) curve ranging from 0.689 to 0.738, compared to qSOFA score of 2 or higher alone [9]. Moreover, the integration of procalcitonin into the qSOFA score has demonstrated modestly useful predictive diagnostic accuracy for nonsevere community-onset bacteremia [10]. The inclusion of predictors that are readily accessible in routine clinical practice could potentially augment the predictive accuracy of qSOFA in diagnosing bacteremia, without adding complexity.

Physical activity helps in recovery in older patients with acute illnesses [11,12]. The Eastern Cooperative Oncology Group-Performance Status (ECOG-PS) is a straightforward scale that assesses a patient's daily physical abilities on a five-point scale. It demonstrates relatively high reliability in comparison to alternative performance status measurement tools [13,14]. Despite its initial development for cancer patients, ECOG-PS has gained widespread utilization across diverse patient populations [15]. For example, performance status was independently associated with the prediction of bacteremia [16]. Furthermore, ECOG-PS score of more than 2 is possibly indicative of an increased risk for bloodstream infection in ICU patients [17]. However, to date, studies investigating the use of ECOG-PS and qSOFA score together for the prediction of bacteremia in older patients with suspected infections have not been conducted. This study aimed to assess the predictive value of augmenting the qSOFA score with the ECOG-PS score in forecasting bacteremia among older patients with suspected infectious diseases.

## Materials and methods

Study design

This prospective observational study took place at the Department of General Medicine within Iizuka Hospital, a 1,048 bed capacity acute care teaching hospital located in Fukuoka, Japan. The study adhered to the principles delineated in the Declaration of Helsinki and conformed to the Ethical Guidelines for Epidemiological Research in Japan. Ethical clearance for the study was obtained from the ethics committee of Iizuka Hospital, and the assigned approval number was 17135. Stringent adherence to the Standards for Reporting of Diagnostic Accuracy Studies (STARD) guidelines was observed, ensuring the transparent reporting of our research [18].

Patients

We consecutively enrolled patients aged ≥65 years, admitted with a suspected infection of bacteremia between January 2018 and 2019. A patient displaying signs of infection underwent a minimum of two sets of blood culture examinations within initial 24 hours of being admitted to the Department of General Medicine [19-24]. The decision to collect blood samples was left to the discretion of patients' healthcare providers.

Measurements

Utilizing a systematically designed data collection form, the researchers gathered information from the electronic medical records of Iizuka Hospital.

Quick Sequential Organ Failure Assessment (qSOFA)

The qSOFA score covers a scale ranging from 0 to 3, assigning one point for each of the specified criteria - hypotension (systolic blood pressure of 100 mmHg or less), tachypnea (respiratory rate of 22 cycles/min or higher), and altered mentation (Glasgow Coma Scale {GCS} less than 15) [1].

Eastern Cooperative Oncology Group-Performance Status (ECOG-PS)

The ECOG-PS score exhibits a range spanning from 0 to 4 [15]. ECOG-PS grade 0 is characterized as "fully active, capable of maintaining all pre-disease performances without limitations;" grade 1 as "limited in physically strenuous activity but ambulatory, and able to engage in light or sedentary tasks (such as light housework and office work);" grade 2 as "ambulatory and capable of all self-care but unable to perform any work activities, being up and about more than 50% of waking hours;" grade 3 as "capable of only restricted self-care, confined to bed or chair for more than 50% of waking hours;" and grade 4 as "completely disabled, unable to carry out any self-care, entirely confined to bed or chair." The Japanese iteration of the ECOG-PS was acquired from the Japan Clinical Oncology Group website [25]. ECOG-PS scores were determined by attending physicians through interviews conducted with patients or their caregivers at the time of admission.

Outcomes

A minimum of two sets of blood cultures, one each for aerobic and anaerobic blood cultures, were systematically collected from all the patients within the initial 24 hours following admission. BACTEC (Sparks, MD: Becton Dickinson) was used as the method of choice for blood culture at Iizuka Hospital. The prescribed minimum incubation period for these cultures was seven days. It should be emphasized that a positive blood culture is not always suggestive of bacteremia, and could be a result of contamination by common skin pathogens [26]. Therefore, in the context of this study, the diagnosis of bacteremia was restricted to cases where two or more blood cultures were positive for the specific pathogenic organism, designating them as authentic bacteremia. In situations where only one blood culture yielded a positive result (including cases with two or more positive cultures for distinct pathogens), an impartial evaluation was conducted by two infectious disease experts, YY and HI. These assessments were executed in a blinded fashion, with the experts uninformed about details regarding ECOG-PS and shaking chills to prevent potential biases [27]. They were asked to make judgments based solely on the datasheet, which listed only the following other variables and species of bacteria. These specialists based their evaluations on supplementary clinical data, including clinical progression and bacterial strain characteristics. Any discrepancies between the two specialists were resolved through collaborative discussion.

Other variables

Upon admission, an electronic medical records database was utilized to assess the following variables: age, gender, body mass index (BMI), body temperature, diastolic blood pressure, heart rate, comorbidities, immunosuppressive treatment status, and various laboratory data (including white blood cell count, platelet count, C-reactive protein {CRP}, serum albumin, and serum creatinine).

Statistical analysis

Categorical variables were expressed in terms of frequency and percentage. Given that all continuous variables displayed a non-normal distribution, their median and interquartile ranges (IQR) were determined.

In the initial analysis, a comparison was conducted on the baseline characteristics of patients with positive and negative blood culture tests. For the comparison of continuous variables between the two groups, p-values were calculated using the Mann-Whitney U test. In the analysis of categorical variables between the two groups, p-values were ascertained using the χ^2^ test, or Fisher's exact test in instances where the count in any category was five or fewer. Following this, the computation of sensitivity (Sn), specificity (Sp), positive predictive values (PPVs), negative predictive values (NPVs), positive likelihood ratios (LR+), and negative likelihood ratios (LR-) for predicting bacteremia involved the use of qSOFA and ECOG-PS scores. Cutoff points of 2 for qSOFA and 3 for ECOG-PS were employed, as established in the existing literature [1,28].

Subsequently, we conducted a comparative analysis of the predictive capabilities of two logistic regression models for diagnosing bacteremia. In Model 1, only the qSOFA score was utilized, whereas in Model 2, ECOG-PS was incorporated into the qSOFA model. To evaluate the performance of the models, we generated a ROC curve and examined the area under the curve (AUC).

In the computation of qSOFA (n=136), we employed multiple imputations using chained equations to address missing data [29]. Ten imputed datasets were generated and analyzed independently, with the results subsequently combined utilizing Rubin’s rules [30]. We used Stata version 17.0 (College Station, TX: Stata Corp.) for all analyses. Statistical significance was set at p<0.05.

## Results

Characteristics

Out of the 1,114 patients included in the study, eight individuals (0.72%) necessitated intensive care unit treatment, and a diagnosis of bacteremia was established in 221 cases (19.8%). Table 1 provides a comprehensive overview of the characteristics of the patients included in the study. The age of the enrolled patients was represented by the median (IQR) of 83 (75-89) years, with 602 (54.0%) of them being female. Notably, individuals with bacteremia exhibited elevated temperatures, more frequent shaking chills, and higher heart rates compared to their counterparts without bacteremia.

**Table 1:** Characteristics of enrolled patients. IQR: interquartile range; BMI: body mass index

Variables	Patients without bacteremia (n=893)	Patients with bacteremia (n=221)	Data missing	p-Value
Sex (male/female)	419/474	93/128	0	0.112
Age (years), median (IQR)	82 (75-89)	82 (76-89)	0	0.593
Shaking chills, n (%)	19 (2.1)	41 (18.6)	3	0.000
Indwelling vascular catheter, n (%)	10 (1.1)	5 (2.3)	0	0.159
Consciousness disturbance, n (%)	369 (41.3)	96 (43.4)	2	0.319
Diabetes mellitus, n (%)	240 (26.9)	61 (27.6)	0	0.444
Immunosuppression drugs, n (%)	18 (2.0)	5 (2.3)	0	0.536
BMI (kg/m^2^), median (IQR)	20.2 (17.7-22.5)	20.6 (17.8-23.2)	2	0.243
Body temperature (°C), median (IQR)	37.2 (36.5-37.9)	38.0 (37.2-39.2)	1	0.000
Systolic blood pressure (mmHg), median (IQR)	129 (108-149)	124 (102-143)	10	0.014
Heart rate (/min), median (IQR)	91 (76-103)	98 (82-108)	3	0.000
Respiratory rate (/min), median (IQR)	22 (18-24)	20 (18-24)	127	0.083
White blood cell count (/μL), median (IQR)	10,646 (6,790-13,300)	12,614 (7,840-15,320)	0	0.001
C-reactive protein (mg/dL), median (IQR)	8.4 (1.9-12.5)	10.1 (3.0-14.4)	1	0.001

Predictive performance of qSOFA and ECOG-PS scores

In the overall cohort, 290 (26.0%) patients had a qSOFA score of 0, 427 (38.3%) patients had a score of 1, 211 (18.9%) patients had a score of 2, and 50 (12.2%) patients had a score of 3. Using a qSOFA cutoff point of ≥2, the sensitivity (Sn) of qSOFA was 32.5% (95% confidence interval, CI: 25.9-39.6), while the specificity (Sp) was 74.7% (71.6-77.8) [1]. The positive and negative likelihood ratios (LR+ and LR-) were 1.29 (1.02-1.63) and 0.90 (0.81-1.00), respectively. The positive predictive value (PPV) was 24.1% (19.1-29.8), and the negative predictive value (NPV) was 81.7% (78.7-84.5) (Table 2). Comparatively, with a qSOFA score of 0 as the reference, the odds ratios (OR) for qSOFA scores of 1, 2, and 3 were determined as 0.89 (95% CI: 0.61-1.31), 1.30 (0.84-2.00), and 1.47 (0.73-2.94), respectively.

**Table 2:** Summary of pooled estimates of qSOFA for the prediction of bacteremia. qSOFA: quick Sequential Organ Failure Assessment; LR+: positive likelihood ratio; LR-: negative likelihood ratio; PPV: positive predictive value; NPV: negative predictive value; 95% CI: 95% confidence interval; LR: likelihood ratio

Cutoff	Total (n=1114)	Bacteremia (n=221)	Sensitivity (95% CI)	Specificity (95% CI)	LR+ (95% CI)	LR- (95% CI)	PPV (95% CI)	NPV (95% CI)
≥1	688	138	72.1% (65.7-77.9)	29.9% (26.9-33.0)	1.03 (0.94-1.13)	0.94 (0.74-1.18)	20.3% (17.5-23.3)	81.2% (76.6-85.3)
≥2	261	63	33.8% (27.6-40.5)	74.6% (71.6-77.4)	1.33 (1.07-1.65)	0.89 (0.80-0.98)	24.8% (20.0-30.1)	82.0% (79.2-84.6)
≥3	50	13	7.2% (4.2-11.5)	95.5% (94.0-96.8)	1.64 (0.93-2.87)	0.97 (0.93-1.01)	28.8% (17.4-42.5)	80.6% (78.1-83.0)

In the overall cohort, 190 (17.1%) patients had an ECOG-PS score of 0, 211 (18.9%) patients had a score of 1, 184 (16.5%) patients had a score of 2, 294 (26.4%) patients had a score of 3, and 235 (21.1%) patients had a score of 4. Utilizing an ECOG-PS score ≥3 as the threshold, the sensitivity (Sn) of ECOG-PS was determined as 48.4% (95% CI: 41.7-55.2), with a corresponding specificity (Sp) of 52.7% (49.4-56.1) [28]. The positive and negative likelihood ratios (LR+ and LR-) were 1.02 (0.88-1.19) and 0.98 (0.85-1.13), respectively. The positive predictive value (PPV) and negative predictive value (NPV) were 20.2% (16.9-23.9) and 80.5% (77.1-83.6), respectively (Table 3). When comparing to an ECOG-PS score of 0 as the baseline, the odds ratios (OR) for ECOG-PS scores of 1, 2, 3, and 4 were calculated as 1.2 (95% CI: 0.75-1.97), 0.77 (0.45-1.32), 1.06 (0.67-1.68), and 1.03 (0.64-1.67), respectively.

**Table 3:** Summary of pooled estimates of ECOG-PS for the prediction of bacteremia. ECOG-PS: Eastern Cooperative Oncology Group performance; LR+: positive likelihood ratio; LR-: negative likelihood ratio; PPV: positive predictive value; NPV: negative predictive value; 95% CI: 95% confidence interval; LR: likelihood ratio

Cutoff	Total (n=1114)	Bacteremia (n=221)	Sensitivity (95% CI)	Specificity (95% CI)	LR+ (95% CI)	LR- (95% CI)	PPV (95% CI)	NPV (95% CI)
≥1	924	184	83.3% (77.7 -87.9)	17.1% (14.7 -19.8)	1.00 (0.94-1.07)	0.98 (0.70-1.36)	19.9% (17.4-22.6)	80.5% (74.2-85.9)
≥2	713	136	61.5% (54.8 -68.0)	35.4% (32.2 -38.6)	0.95 (0.85-1.07)	1.09 (0.90-1.31)	19.1% (16.3-22.2)	78.8% (74.5-82.7)
≥3	529	107	48.4% (41.7 -55.2)	52.7% (49.4 -56.1)	1.02 (0.88- 1.19)	0.98 (0.85-1.13)	20.2% (16.9- 23.9)	80.5% (77.1- 83.6)
≥4	235	47	21.3% (16.1 -27.3)	78.9% (76.1 -81.6)	1.01 (0.76-1.34)	1.00 (0.92-1.08)	20.0% (15.1-25.7)	80.2% (77.4-82.8)

Predictive performance of Model 1 and Model 2

**Figure 1:**
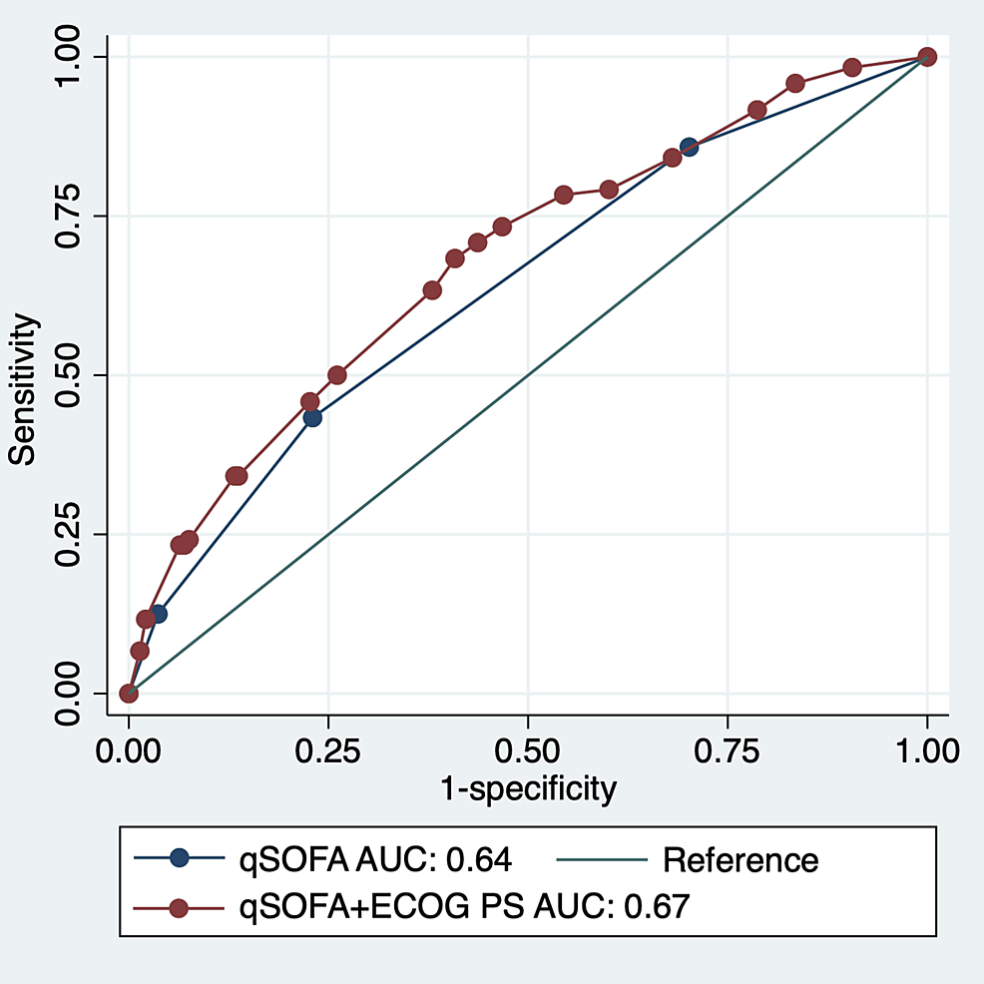
Receiver operating characteristic curves, illustrating the predictive efficacy of Model 1 and Model 2 in forecasting bacteremia. Receiver operating characteristic (ROC) curves delineate the models' capacity for predicting bacteremia within older patients. AUC: area under the receiver operating characteristic curve; qSOFA: quick Sequential Organ Failure Assessment; ECOG-PS: Eastern Cooperative Oncology Group-Performance Status

The formulae for the Model 1 and Model 2 are shown in Table 4 and Table 5, respectively. The AUC of Model 2 was not significantly different from the qSOFA model (0.54, 95% CI: 0.5-0.59 vs. 0.55, 95% CI: 0.5-0.59; p=0.162) (Figure 1). In our study, we presented the coefficient and standard error for each predictor in the predictive model. This dual inclusion is pivotal for demonstrating the model's accuracy and reliability. The coefficient provides an estimated impact of each predictor on the outcome, a key aspect for pinpointing influential variables in the prediction. Meanwhile, the standard error gauges the precision of these coefficients, reflecting our confidence in the estimates and their consistency across various samples. Incorporating these metrics not only underpins the validity of our model but also aids in comparing and replicating our results in future research, thereby bolstering the study's credibility and its practical relevance in diverse clinical contexts.

**Table 4:** Prediction of bacteremia defined by logistic regression analysis of Model 1. qSOFA: quick Sequential Organ Failure Assessment

	Coefficient	Standard error
qSOFA		
1	-0.10	0.18
2	0.30	0.21
3	0.52	0.33
Constant	-1.45	0.14

**Table 5:** Prediction of bacteremia defined by logistic regression analysis of Model 2. qSOFA: quick Sequential Organ Failure Assessment; ECOG-PS: Eastern Cooperative Oncology Group-Performance Status

	Coefficient	Standard error
qSOFA		
1	-0.08	0.19
2	0.33	0.21
3	0.55	0.34
ECOG-PS		
1	0.19	0.25
2	-0.29	0.28
3	0.01	0.24
4	-0.05	0.25
Constant	-1.46	0.21

## Discussion

This study was conducted to determine whether adding the ECOG-PS score to the qSOFA score improves the accuracy of predicting bacteremia in older patients with suspected infections. Our research found that including the ECOG-PS score with the qSOFA score did not significantly improve the predictive accuracy for bacteremia.

This study confirmed that the qSOFA score is a simple clinical tool measuring blood pressure, respiratory rate, and consciousness level, and has limited predictive performance for the diagnosis of bacteremia in older patients with infections. Interestingly, contradictory to previous studies, our study demonstrated that the performance of qSOFA in diagnosing bacteremia was inadequate [31]. This inconsistency could be because our study included a higher proportion of older patients than the previous studies, who by the virtue of their age might experience sudden imbalances in the body due to several serious medical conditions [29]. Hence, relying solely on the qSOFA score might not be sufficient to identify patients with bacteremia [29]. Healthcare providers must recognize the limitations of the qSOFA when attempting to diagnose bacteremia.

Although we hypothesized that combining ECOG-PS with qSOFA scores would improve the predictive accuracy of bacteremia, this combination model did not result in significant improvement. The ECOG-PS was developed to evaluate the performance of daily tasks and physical activity in patients with cancer [15]. Performance status is one of the risk factors for infection. However, bacteremia could be influenced by other risk factors, such as the patients’ immune response and other chronic diseases (e.g., diabetes mellitus, cancer, and chronic kidney disease) [32]. Therefore, the ECOG-PS might not adequately reflect these complexities. It may be prudent to acknowledge the limitations inherent in simplistic scoring systems. In addition to scores used to determine and predict the severity of infectious diseases, ratios such as fibrinogen to albumin ratio are also being investigated [33]. Additionally, artificial intelligence models are being developed to predict the severity of the disease in intensive care units [34]. It may be worth contemplating the adoption of more advanced scoring systems leveraging artificial intelligence methodologies [34].

The study has important practical implications. The study highlights that in older patients, the use of only vital signs and physical function assessments for predicting positive blood cultures for bacteremia is not adequate and accurate. Our results emphasize the importance of conducting thorough patient interviews to determine signs and symptoms (e.g., shivering) and other risk factors (e.g., underlying medical conditions). Further, this study suggests the need for further research to develop more accurate models for predicting bacteremia, specifically in older patients. Collaboration between healthcare institutions and research teams could help identify new variables and tools to improve predictive accuracy.

This study had a few limitations. First, one of the study inclusion criteria was a subjective measure of diagnosis confirmation based on blood culture results by physicians. This could decrease the reproducibility of the study findings. However, this criterion was used based on previous research, and ethical considerations of not repeating blood culture tests on all patients [13-16,18,19]. Second, our study was conducted at a single center. This could limit the generalizability of our findings.

## Conclusions

In summary, our study explored the possibility of using qSOFA and ECOG-PS scores together to predict bacteremia in older patients with suspected infections. Although the use of ECOG-PS along with qSOFA did not significantly improve the predictive accuracy of bacteremia, our results offered important insights into the prediction of infectious diseases in older patients. Further research and clinical validation are necessary to develop effective tools for identifying patients with bacteremia.
